# Exploring Instant Noodle Consumption Patterns and Consumer Awareness in Kosovo

**DOI:** 10.3390/foods14244245

**Published:** 2025-12-10

**Authors:** Salih Salihu, Besjana Elezaj, Dejsi Qorri, Njomza Gashi

**Affiliations:** 1 Department of Food Technology with Biotechnology, Faculty of Agriculture and Veterinary, University of Prishtina, 10000 Prishtina, Kosovo; 2 Department of Urban Agriculture, Faculty of Agriculture and Veterinary, University of Prishtina, 10000 Prishtina, Kosovo; 3 Institute of Economics, Faculty of Economics and Business, University of Debrecen, 4032 Debrecen, Hungary; 4 Center for Complex Systems and Microbiome Innovations, Faculty of Agricultural and Food Sciences and Environmental Management, University of Debrecen, 4032 Debrecen, Hungary

**Keywords:** instant noodles, consumption patterns, nutrition awareness, purchasing decisions, Kosovo

## Abstract

Instant noodles have become a staple convenience food worldwide, with rising consumption particularly among younger, busier populations. This study investigates consumer perceptions, health concerns, consumption habits, and purchasing behaviors related to pre-packaged noodles in Kosovo. A structured questionnaire was administered to 400 participants, exploring attitudes toward health impacts, ingredient awareness, product preferences, and purchasing motivations. Findings revealed mixed perceptions of noodle healthiness, with older and more educated individuals significantly more likely to view them as unhealthy (*p* < 0.001). Although most respondents expressed concern about ingredients such as fat, calories, and salt, awareness of additives like monosodium glutamate (MSG) remained low (*p* < 0.001), indicating a gap in consumer knowledge. Consumption patterns varied notably by age, gender, income, and health status. Younger and lower-income groups consumed noodles more frequently (*p* < 0.001), often driven by time constraints rather than taste or nutritional value. One-third of participants reported a sense of addiction, strongly linked to both frequency and portion size. When purchasing, consumers prioritized label clarity, origin, and natural ingredients over sensory appeal, and advertising exerted only a moderate influence on choices. These results suggest that while practical needs drive consumption, health concerns and demographic factors strongly shape perceptions and behavior. Efforts to reduce unhealthy consumption should focus on clearer labeling, accessible nutrition education, and promoting healthier, convenient alternatives.

## 1. Introduction

Instant noodles are known as a popular food choice across Asian countries, which continue to gain popularity worldwide due to their convenience. Their rapid preparation time, appealing taste, affordability, and extended shelf life make them a preferred choice for many consumers. Key quality attributes include appearance (color), aroma, texture, cooking behavior, water absorption capacity, and shelf stability [1]. In 2024, global consumption reached 123.1 billion servings, with China Hong Kong leading at 43.8 billion servings, followed by Indonesia with 14.7 billion, and India at 8.3 billion, marking these regions as the largest consumers globally [2].

Within Europe, Russia tops the list with 2.29 billion servings, Germany with 430 million, and Poland with 370 million servings. Although precise consumption figures for Kosovo are unavailable, there is evident growth in instant noodle consumption, driven by shifting dietary habits and greater market availability [2].

The primary ingredients of instant noodles typically include flours and starches derived from wheat, maize, potato, buckwheat, barley, rye, soy, or other crops, combined with water and salt. This composition results in a product rich in carbohydrates and fats but generally low in essential nutrients such as fiber, vitamins, and minerals necessary for a balanced diet [3]. To improve nutritional content and texture, instant noodles are often supplemented with protein-enriching additives like soy flour, soy milk, egg yolk, chicken meat, or seaweed. Hydrocolloids, such as xanthan gum, guar gum, alginate, and carboxymethyl cellulose, are added to enhance water retention, texture, and cooking quality, while also lowering the glycemic index. Moreover, enzymes, organic acids (e.g., citric and lactic acids), phytochemicals, and natural colorants like beetroot, turmeric, and red dragon fruit peel contribute to extending shelf life, inhibiting microbial growth, and improving both appearance and functional properties [4]. A widely recognized additive in instant noodles is MSG, a flavor enhancer that amplifies the umami taste. MSG, identified as E621 under European food regulations, is the sodium salt of glutamic acid, an amino acid naturally present in plants and animals [5]. In instant noodles, MSG is utilized to boost flavor intensity and enhance the overall palatability, forming a key component of seasoning mixes and broth bases [6].

Due to their nutritional imbalance, instant noodles are often perceived as an unhealthy dietary choice. The main health concerns linked to their consumption arise from typically high sodium and fat contents, which have been consistently associated with increased risks of hypertension, cardiovascular diseases, and obesity [7]. Regular consumption of such nutrient-poor foods can lead to excessive sodium intake, contributing to elevated blood pressure and other related health issues [8]. Furthermore, instant noodles generally lack adequate levels of essential nutrients, including dietary fiber, vitamins, and minerals vital for maintaining overall health. The common frying process used in their production can also result in the formation of unhealthy trans-fatty acids, which further heighten the risk of metabolic and cardiovascular diseases [7]. Beyond their nutritional composition, instant noodles are widely classified as ultra-processed foods (UPFs) due to the intensive industrial techniques used in their manufacture, a group increasingly debated within nutrition and public health research [9]. The production process typically involves multiple stages such as pre-gelatinization and deep-frying of the dough, dehydration, and the incorporation of seasoning powders and flavoring oils rich in sodium, fats, and additives to enhance texture, taste, and shelf stability [1,10]. These steps substantially alter the original food matrix and create a product engineered for long storage and strong sensory appeal, characteristics that align with the UPF profile [11]. However, their consumption also reflects complex social and economic dimensions, including affordability, accessibility, lifestyle convenience, and cultural adaptation. In many contexts, instant noodles serve as both an economical meal option and a symbol of modernization and globalized eating habits [1]. Given these features, instant noodles represent a relevant case study within discussions on the health implications of processed foods and consumer awareness of nutrition and labeling. However, understanding noodle consumption requires a multidimensional perspective that integrates nutritional, behavioral, and socioeconomic factors, acknowledging that consumer choices are influenced not only by health considerations but also by time constraints, marketing exposure, and cultural preferences [12].

Despite growing concern about their nutritional value instant noodles remain embedded in everyday eating habits, especially among younger and urban populations. This persistence highlights a need to understand how consumers interpret product information, perceive associated health risks, and make purchasing decisions in light of broader public-health messaging. Instant noodles have gained widespread popularity across many regions, primarily due to their convenience, affordability, and wide variety of flavors [13]. These qualities make instant noodles particularly appealing to individuals with hectic lifestyles and limited time for meal preparation. As global dietary habits evolve, instant noodles continue to hold a firm place in many households, especially within urban environments. However, consumption patterns vary significantly depending on factors such as cultural background, income level, and demographic characteristics [14]. Understanding these driving factors is essential to gaining deeper insights into shifting food behaviors, which underscores the importance of examining the motivations behind instant noodle consumption in diverse populations.

While extensive research on instant noodle consumption exists internationally [14,15,16], there is a notable lack of studies focusing on Kosovo. Currently, no official data is available concerning consumption frequency, influencing factors, or consumer perceptions of instant noodles within the Kosovar context. Nonetheless, the abundant availability of various instant noodle brands and types in local stores suggests a growing consumer interest. This variety not only reflects market demand but also indicates potential changes in dietary habits, particularly among younger and urban demographics. This study aims to examine consumer knowledge and perceptions of instant noodles, with an emphasis on nutritional awareness and information processing. By analyzing these factors, it contributes to understanding how awareness, education, and labeling transparency influence everyday food choices. Therefore, this study aims to investigate instant noodle consumption patterns in Kosovo, evaluate consumer knowledge of these products, identify primary information channels, and explore how consumption behavior varies by gender, age, and education levels. The findings are expected to support informed consumer choices and contribute to food-literacy and public-health initiatives addressing ultra-processed food consumption.

## 2. Materials and Methods

### 2.1. Questionnaire Design and Data Collection

The research was based on a structured questionnaire specifically designed to capture consumer perceptions, attitudes, and behaviors related to noodle consumption in Kosovo. According to Yamane’s formula [17] for sample size determination, a total of 400 valid responses were sufficient to ensure a 95% confidence level with a 5% margin of error for Kosovo’s adult population. The questionnaire was self-developed based on established approaches in consumer behavior and food perception research. It comprised 19 closed-ended questions, organized into thematic sections that addressed different aspects of consumer habits, purchasing preferences, and attitudes toward instant noodles. The first section included questions about the frequency and quantity of consumption, assessed through single-choice categorical options, allowing for an assessment of typical intake levels. The second section assessed knowledge and perceptions of pre-prepared noodles using five-point Likert-type scales (1 = strongly disagree to 5 = strongly agree) for statements such as “They are healthy” or “Frequent consumption may have a negative effect on my health”. Additional questions in this section applied yes/no and single-choice formats to assess ingredient awareness and product-related concerns. The third section focused on consumption habits and purchasing preferences, including frequency, portion size, time of consumption, satisfaction, and factors influencing product choice. Most questions were single-choice, except for the question regarding factors influencing the product choice, which allowed multiple responses. The instrument was internally reviewed by the research team to ensure clarity, logical structure, and alignment with the study objectives. The study did not require formal ethical approval, as it was conducted through an anonymous and voluntary questionnaire that collected no personally identifiable information. All participants were informed about the purpose of the research and provided their consent before participation. The questionnaire was distributed online using a voluntary sampling approach, with the link shared via email and social media platforms to reach participants from different regions of Kosovo. Only respondents aged 18 years and above were included in the analysis. Data collection was conducted anonymously, and all responses were automatically recorded and stored in accordance with ethical and data protection standards. To guarantee privacy and compliance with ethical standards, no personal identifiers were collected, and all data were anonymized in accordance with the European Union’s General Data Protection Regulation (Regulation (EU) 2016/679) [18].

### 2.2. Statistical Analysis

Data generated from the questionnaires were analyzed using IBM SPSS Statistics (version 25) and RStudio software (version 4.4.0). All analyses were performed on the full dataset (n = 400). Descriptive statistics, including frequency distributions and percentages, were computed in SPSS to summarize the demographic characteristics of the respondents and to describe consumption patterns. Given that most variables were categorical or ordinal in nature, non-parametric tests were selected without performing formal normality testing. Specifically, Chi-square tests were used to examine relationships between categorical variables, the Mann–Whitney U test to compare differences between groups, and Spearman’s rank correlation coefficient to assess associations between continuous or ordinal variables. All statistical tests were performed at a 95% confidence level (*p* < 0.05). In addition to statistical testing, a range of graphical representations was created to enhance the clarity and interpretability of results. Heatmaps were used to visualize associations between categorical variables, including demographic factors and MSG awareness, as well as the percentage distribution of consumption patterns across gender, age, education, and income groups. Corresponding Multidimensional Scaling (MDS) plots illustrated similarities and clustering tendencies among respondents based on these demographic variables. A circular bar chart summarized the frequency of pre-packaged noodle consumption, while donut charts depicted detailed aspects of consumption behavior such as the number of packages per meal, perceived dependency, time of consumption, and accompanying beverages. Bar plots were used to present levels of consumer satisfaction and the relative importance of factors influencing purchasing decisions, including the role of advertising. Visualizations were primarily developed in RStudio (2025.09.0+387) using packages such as ggplot2 (for bar plots and donut charts), ggpubr (for enhancing statistical plots), ComplexHeatmap (for heatmaps), and vegan (for ordination analyses such as MDS plots).

### 2.3. Respondent Characteristics

The demographic characteristics of the respondents are presented in Table 1. Among the 400 participants, females constituted the majority (70.5%), while males accounted for 29.5%. The largest age group was 18–24 years (32.5%), followed by 25–34 years (24.3%), and under 18 years (12.0%). Regarding educational attainment, over half of the respondents held a bachelor’s degree (53.8%), while 21.3% had completed high school, 20.8% held a master’s degree, 3.0% had a PhD, and only 1.3% had a middle school education. In terms of income distribution, the majority reported earning between 901–1200 euros (43.2%), followed by those with incomes above 1200 euros (25.7%) and between 601–900 euros (23.3%). Only a small proportion reported incomes below 600 euros. These data highlight that the sample predominantly consisted of young, highly educated individuals with mid-to-high income levels.

## 3. Results

### 3.1. Perceptions of Pre-Packaged Noodles

The perception of pre-packaged noodles varied considerably among respondents (Table 2). Nearly half of the participants remained neutral regarding whether such products are healthy (45.2%), while almost one-quarter disagreed (23.2%). This perception did not differ by gender but showed apparent variation by education level (*p* < 0.001). Moreover, age was negatively correlated with this view (Spearman’s ρ = −0.40, *p* < 0.001). When asked to compare with traditional cooking, half of the respondents (50%) agreed that pre-packaged noodles represent a less healthy alternative. This view varied significantly by education (*p* < 0.001) and was positively correlated with age (*p* < 0.001).

Perceptions of salt content were more mixed. While most participants expressed neutrality (42.8%), one-quarter disagreed (25.4%). Gender differences were significant (*p* = 0.001), as were differences by education (*p* < 0.001). Age again showed a strong negative correlation (ρ = −0.44, *p* < 0.001). Opinions on the potential for weight gain also varied. Although 37.2% of respondents were neutral, nearly one-third (32%) agreed that pre-packaged noodles contribute to weight gain. These views differed significantly across education levels (*p* < 0.001) and were positively correlated with age (ρ = 0.36, *p* < 0.001), while gender differences were not significant (*p* = 0.55). Clarity of ingredient lists was viewed more positively. Almost half of the participants (43.8%) agreed that the labeling was clear. Responses differed by education (*p* = 0.001), while no statistically significant differences were observed by gender (*p* = 0.06). A small but significant negative correlation with age was also detected (ρ = −0.16, *p* = 0.001).

### 3.2. Knowledge and Health Concerns

In addition to perceptions, participants reported various health concerns and levels of knowledge regarding the nutritional profile and additives in pre-packaged noodles. Concerns about health impacts were widespread. A majority believed that frequent consumption may negatively affect health (47.4% agree, 15.2% strongly agree). This perception varied significantly by education (*p* < 0.001) and was positively associated with age (ρ = 0.29, *p* < 0.001), (Table 3). When asked about specific health concerns, participants primarily pointed to the nutritional profile of noodles. The most frequently reported issues were high fat and calorie content (37.1%) and the use of additives and preservatives (32.1%). Concerns about salt were also notable (20.4%) and showed significant associations with gender (χ^2^ = 11.21, *p* = 0.011), education (χ^2^ = 48.29, *p* < 0.001), and especially age (χ^2^ = 134.63, *p* < 0.001). Only a small proportion (10.5%) reported having no concerns at all.

**Figure 1:**
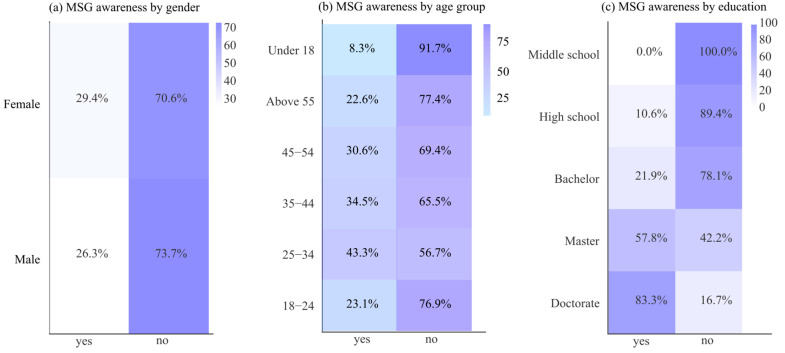
Associations of MSG awareness with demographic factors: (**a**) gender, (**b**) age group, and (**c**) education level.

Awareness of MSG was relatively low (Table 4). Only 28.5% of respondents reported knowing that noodles may contain MSG, while the majority (71.5%) were unaware. No significant gender differences were observed (*p* = 0.523), although females tended to be slightly more informed. In contrast, education (χ^2^ = 72.78, *p* < 0.001) and age (χ^2^ = 23.51, *p* < 0.001) were both strongly associated with awareness, with more educated and middle-aged individuals more likely to recognize the presence of MSG (Figure 1).

### 3.3. Consumption Behavior of Pre-Packaged Noodles

**Figure 2:**
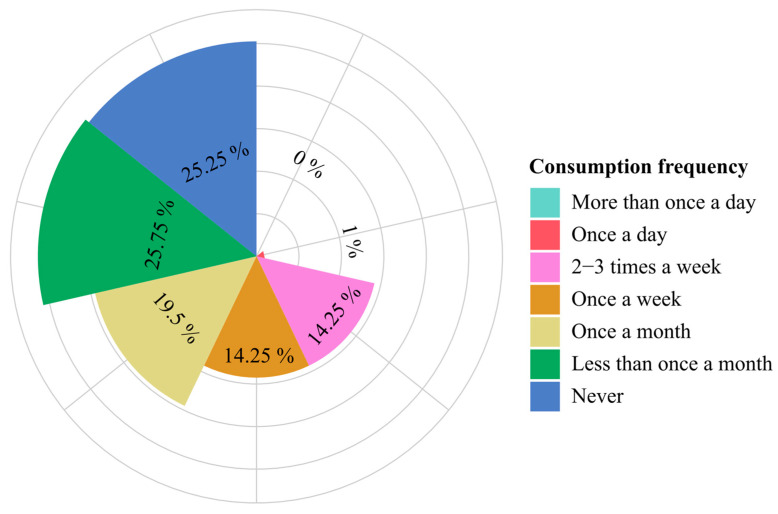
Circular bar chart showing the distribution of pre-packaged noodle consumption frequency.

The overall frequency of noodle consumption among respondents showed that most participants ate noodles less than once a month (25.75%) or never (25.25%), while very few reported daily consumption (Figure 2).

**Figure 3:**
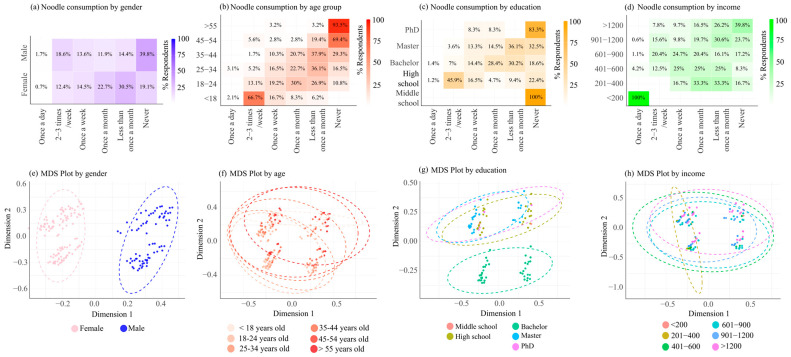
Visualization of instant noodle consumption across demographic groups. Subfigures (**a**–**d**) present heatmaps showing the percentage distribution of consumption by gender (**a**), age group (**b**), education level (**c**), and income level (**d**). Subfigures (**e**–**h**) display the corresponding Multidimensional Scaling (MDS) plots for gender (**e**), age (**f**), education (**g**), and income (**h**), highlighting patterns and similarities in consumption behavior within each demographic category.

When examined by gender, males were more likely to report never consuming noodles (39.8%), whereas females more often consumed them less than once a month or once a month (Figure 3). Age-based differences were also evident: the youngest group (<18 years) had the highest consumption, particularly 2–3 times per week (66.7%), while the oldest group (>55 years) mostly reported never consuming noodles (93.5%). Education and income also influenced consumption patterns. Individuals with higher education levels (master’s and PhD) tended to consume noodles less frequently, mostly once a month or less. Similarly, respondents in the lowest income bracket (<200) showed the highest consumption, whereas those with higher incomes reported lower rates, indicating that noodles may be perceived as a more affordable option. MDS plots further illustrated these differences, with gender-based clustering showing the clearest separation, reflecting distinct consumption habits between males and females.

**Figure 4:**
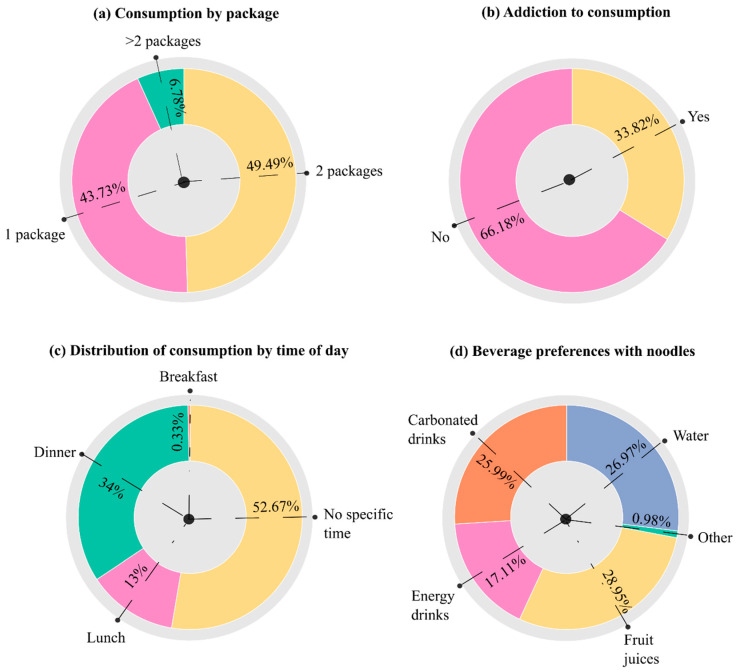
Donut charts showing: (**a**) packs per meal, (**b**) consumption dependency, (**c**) time of consumption, and (**d**) accompanying drinks.

To provide a comprehensive view of packaged noodle consumption, the analysis included portion size, measured as the number of packages consumed per meal, along with perceived addiction, timing of consumption, and beverage preferences (Figure 4). Most participants consumed one (43.73%) or two packages (49.49%), with only a small proportion (6.78%) consuming more than two, indicating generally moderate intake. Approximately one-third (33.82%) reported feeling addicted, while the majority (66.18%) did not perceive themselves as dependent. Regarding consumption timing, more than half of respondents (52.67%) had no fixed schedule, reflecting irregular or opportunistic intake. Among those with defined times, dinner was the most common, followed by lunch, while breakfast was rarely reported. When paired with noodles, beverage preferences were diverse. Fruit juices were most commonly chosen, followed by water and carbonated drinks, while energy drinks were selected by 17.11%.

Finally, the analysis of reasons for consumption revealed that the predominant motivator was lack of time to prepare meals, reported by 75.17% of respondents (Table 5). This reason was strongly associated with gender (χ^2^ = 33.629, *p* < 0.001) and age (χ^2^ = 60.550, *p* < 0.001). Other reasons, such as not knowing how to cook or convenience during breaks, were less frequently reported. Perceived addiction was significantly associated with both consumption frequency (χ^2^ = 321.773, *p* < 0.001) and the number of packages consumed (χ^2^ = 194.557, *p* < 0.001). Individuals who consumed noodles more often or in larger quantities were more likely to report a sense of habitual intake (Table 6).

### 3.4. Factors Influencing Noodle Purchases

**Figure 5:**
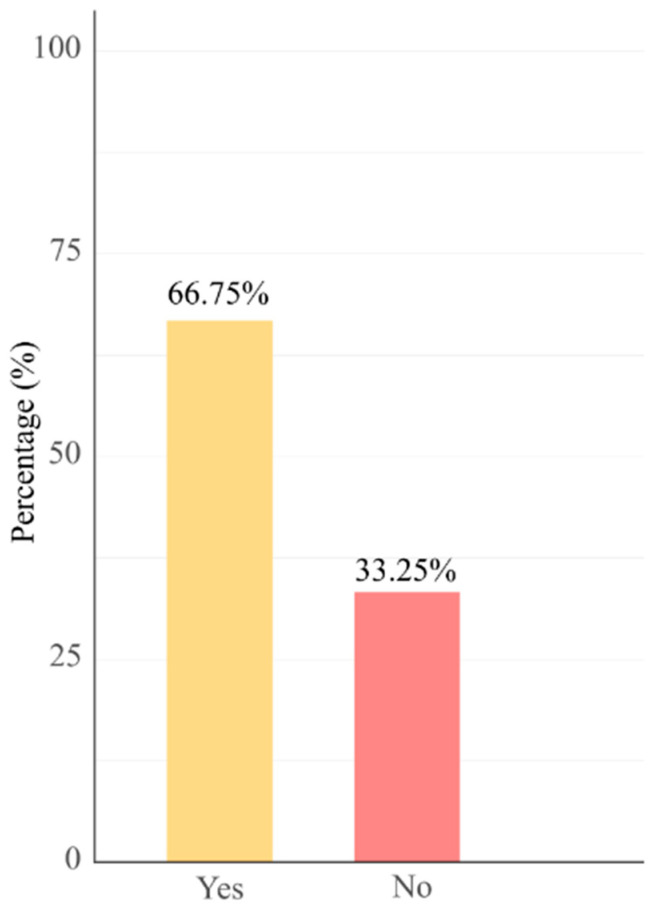
Consumer satisfaction with instant noodles available in markets across Kosovo.

Since consumer choices are shaped not only by product characteristics but also by marketing strategies, it is important to examine the factors that influence noodle purchases. The majority of respondents reported being satisfied with the quality of noodles available in the Kosovo market, although 33.25% expressed dissatisfaction (Figure 5).

**Figure 6:**
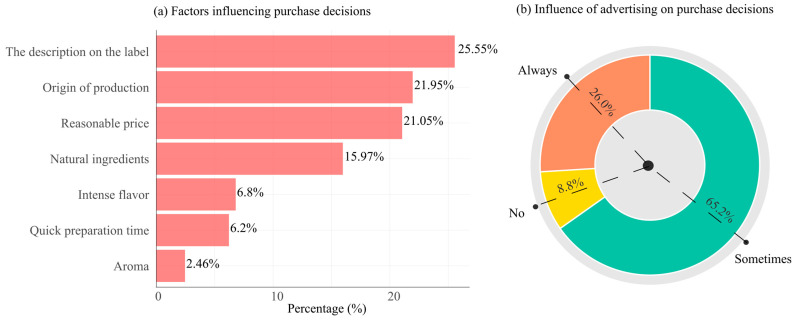
Factors influencing the selection of instant noodles for purchase (**a**) and the impact of advertising on product choice (**b**).

Regarding purchasing decisions, the description on the label emerged as the most decisive factor, followed by the origin of production and reasonable price. Natural ingredients also played a considerable role, whereas sensory attributes such as intense flavor, quick preparation time, and aroma were less influential (Figure 6).

Advertising was found to have a moderate influence on purchasing decisions. Most respondents (65.2%) reported that advertising sometimes affects their choice, 26.0% and only 8.8% stated that it has no effect. This pattern highlights that while advertising contributes to consumer behavior, its impact is not absolute and interacts with other important factors, including labeling, price, and origin of production.

## 4. Discussion

This study aimed to explore consumer perceptions, knowledge, and consumption behaviors related to instant noodles. The results provide valuable insights into how demographic factors shape attitudes toward health, nutrition, and purchasing decisions.

Findings showed that perceptions of instant noodles were often neutral, particularly regarding their healthiness and salt content. However, older and more educated respondents expressed greater concern about potential health impacts such as high sodium intake and weight gain, while gender differences were minimal. Although ingredient labels were generally perceived as clear, older individuals were less likely to find them understandable. These patterns point to a need for improved label design and more targeted health education, especially for older adults and those with lower education levels. This aligns with the findings of Bolhuis et al. [19], who reported that neutral attitudes toward processed foods are often linked to limited nutritional knowledge unless consumers are well informed. According to Raghupathi & Raghupathi [20], both higher education and age are associated with increased awareness of health risks due to better nutritional literacy and heightened sensitivity to chronic disease vulnerability. The difficulty that older adults face in understanding food labels is also supported by FDA guidelines [21], which emphasize that standard labels often use small fonts and technical language that are not senior friendly [22]. Furthermore, the widespread neutrality in perceptions may reflect the cultural normalization of instant noodles as convenient and familiar foods, a phenomenon previously observed by Xiao et al. [23]. Additionally, concern about the health risks of instant noodles was particularly evident among older and more educated individuals, confirming Mancone’s [24] argument that education enhances food literacy and the ability to self-regulate eating habits. While gender did not significantly influence overall concern, females showed more sensitivity to salt intake, in line with findings by Kiefer et al. [25], who reported that women are generally more attentive to nutrition and more likely to adjust their diets for health reasons. These patterns highlight the importance of strengthening consumer food literacy and targeted health education, particularly among younger and lower-educated groups, to reduce misinformation about processed foods and promote healthier choices. Similar studies highlight how experiential, culturally adapted interventions, centered on hands-on learning and community engagement, can effectively enhance adolescent food literacy and promote healthier dietary behaviors across European contexts [26,27]. Moreover, the growing demand for healthier options suggests opportunities for product reformulation and clearer communication strategies aligned with broader European goals for sustainable and health-oriented food systems [28,29].

Concerns about high fat, calorie content, and the use of additives reflect widespread public skepticism toward processed foods, showing broader consumer perceptions that such products are inherently unhealthy. This is consistent with Ciobanu et al. [30], who noted that food additives are often linked by consumers to adverse health effects. Awareness of MSG was relatively low among respondents, a finding similar to that of Sivaharini & Ganapathy [31], who attributed such gaps in knowledge to unclear labeling and inconsistent messaging from both media and health authorities. MSG may not be easily identified by consumers due to its appearance under alternative names on ingredient lists, or because labeling does not clearly emphasize its presence. As a result, many individuals may unknowingly consume MSG, which raises concerns about transparency in food labeling. This lack of clear information limits informed consumer choice and highlights a broader issue in processed food regulation.

Regarding consumption frequency, the data reveal relatively low intake of instant noodles overall, with most participants reporting infrequent or no consumption. Age emerged as a key factor. Younger individuals, particularly those under 18, showed the highest levels of consumption, whereas older adults reported much lower intake. This pattern is likely influenced by increased health awareness and dietary restrictions among older populations, who often avoid processed, high-sodium foods due to risks related to hypertension and weight gain. These results are consistent with Chung et al. [32], who found that noodle consumption was highest among youth aged 9–18 and declined progressively with age. In addition to health concerns, socio-cultural and lifestyle factors also contribute to age-related differences. Younger consumers often prioritize convenience, cost-effectiveness, and time-saving aspects of instant noodles, whereas older individuals may be more concerned with nutrition and long-term health. This supports findings from recent reviews on consumer behavior, which suggest that younger populations are more inclined to choose processed foods for practical reasons, while older groups demonstrate greater willingness to pay for healthier alternatives [33]. Thus, noodle consumption patterns appear to reflect a complex interplay of age, lifestyle, and health literacy. These demographic and perceptual differences can also be interpreted within the context of Kosovo’s broader socioeconomic and cultural transition. Younger, urban populations are more exposed to globalized food markets and fast-paced lifestyles, while older adults remain more attached to traditional, home-cooked meals. Similar generational contrasts have been observed across the Western Balkans, where modernization and market liberalization have accelerated dietary westernization [34]. In this sense, consumer attitudes toward instant noodles in Kosovo mirror broader regional trends in which convenience foods coexist with traditional dietary norms. According to Milošević et al. [35], food choice motives in Western Balkan countries, including factors like convenience, sensory appeal, and health, show similar patterns across the region reflecting broader socioeconomic transitions.

Gender differences in instant noodle consumption were evident, with males more likely to report never eating noodles, while females tended to consume them occasionally. This may reflect broader gender-based differences in dietary habits and health consciousness. Existing research shows that women often consume instant noodles more frequently but may also be more susceptible to associated health risks, such as metabolic syndrome, due to a combination of biological factors and lifestyle behaviors [36]. Socioeconomic status also played a significant role. Individuals with higher levels of education and income generally consumed noodles less frequently, likely reflecting greater health awareness and access to diverse food options. Nonetheless, because most respondents were university students or degree holders, education-related differences should be interpreted cautiously. In contrast, higher consumption among lower-income participants suggests that instant noodles are viewed as a practical and affordable choice. This aligns with findings from other studies, where consumption patterns were shown to be influenced by affordability and accessibility [13,37].

Portion sizes were generally moderate, as nearly all participants reported consuming one or two packages per meal (Figure 4a), while only a small proportion (6.78%) consumed more than two packages. Notably, about one-third of respondents reported feeling addicted to instant noodles, and this sense of dependency was strongly associated with both higher consumption frequency and larger portions. These results suggest a behavioral or emotional component to noodle consumption, where individuals may use noodles as a comfort food or habitual meal rather than due to physical craving. The lack of a fixed consumption schedule for most participants, with dinner being the most common time when noodles were eaten, reinforces this interpretation. These findings are consistent with Babcock et al. [38], who also found irregular noodle consumption patterns among university students. This irregular, opportunistic consumption pattern suggests that many people turn to noodles when they are short on time or energy, often in the evening after a busy day.

Beverage choices paired with noodles ranged from healthier options like water and fruit juice to less healthy ones such as carbonated and energy drinks, reflecting a broad spectrum of dietary behaviors among consumers. These preferences highlight varying levels of health awareness within the population. The primary reason for noodle consumption was lack of time to prepare meals, particularly among younger and working-age groups, underscoring the strong influence of convenience on dietary decisions. This finding aligns with Bogard [39], who emphasized that convenience is a key dimension shaping modern food environments. Other reasons, such as limited cooking skills or the need for quick meals during breaks, further point to the role of time pressure over nutritional considerations. These findings reinforce that instant noodle consumption is driven by a combination of lifestyle demands, personal habits, and demographic characteristics. Therefore, strategies to curb excessive consumption should focus on promoting healthier, convenient alternatives and improving awareness, particularly among younger and lower-income populations.

The findings reveal that while most consumers in Kosovo are generally satisfied with the quality of instant noodles available, a notable 33.25% expressed dissatisfaction, indicating opportunities for improving product quality or consistency. These issues may relate to ingredient standards or alignment with local taste preferences. When making purchase decisions, consumers placed greater emphasis on informational attributes such as label descriptions, product origin, and price, over sensory characteristics like flavor or aroma. This trend reflects a growing demand for transparency and product credibility, consistent with prior research demonstrating that consumers increasingly prioritize health, safety, and quality information above immediate sensory appeal [40].

Natural ingredients emerged as another important factor, underscoring the rising consumer interest in healthier food options. Despite of the common marketing emphasis on quick preparation time, this attribute ranked lower in importance, suggesting that convenience is not the sole driver behind purchasing decisions. Advertising had a moderate influence. While it impacted many consumers occasionally (65.2%), it was rarely the primary motivator. As suggested by Tarigan et al. [41] young consumers tend to be drawn to product innovation, visually attractive presentations, competitive prices, and digital marketing approaches delivered through social media. Recent findings display the importance of strategies such as positioning and product differentiation in consumer loyalty of instant noodles [42]. This suggests that clear labeling (brand, certification, ingredients etc.) combined with quality assurance can be effective marketing strategy, as study suggest that brand awareness and sales promotion in instant noodles have a positive influence in purchase intention, rather than taste, packaging, pricing, and convenience [43]. Overall, these results highlight that consumers value transparency, safety, and product quality, and are not easily swayed by advertising or sensory appeal alone. Recent findings show the important role that brand trust has as a mediator between brand image and customer loyalty [44]. This furthers the key role producers and manufacturers operating in instant noodle industry have in addressing further improvements in labeling clarity, sourcing transparency, and the use of natural ingredients to meet consumer expectations and foster trust, that expands their market share in Kosovo and neighboring countries.

## 5. Conclusions

The findings highlight that most consumers perceive instant noodles as a less healthy alternative to home-cooked meals, mainly due to concerns about salt content and additives. While younger respondents tend to consume instant noodles more frequently because of convenience and lifestyle factors, higher nutritional awareness was more evident among older and more educated participants, indicating that demographic factors influence perceptions and purchasing choices. Although convenience remains an important motivator, consumers increasingly value product transparency, ingredient clarity, and natural content. This shift presents both challenges and opportunities for producers, who must improve labeling practices, ensure consistent product quality, and adapt to evolving consumer expectations. Tailored communication and educational efforts are essential to address knowledge gaps, particularly among older and less informed groups. Future research should further explore strategies to strengthen nutrition literacy and evaluate healthier product innovations that balance quality, affordability, and taste. Policymakers and industry stakeholders should collaborate to promote labeling transparency and consumer education, fostering a more informed and health-conscious market. While the sample composition limits broad generalization, the study offers valuable insight into emerging consumer behaviors in Kosovo and provides a foundation for future comparative and market-oriented research.

Limitations: This study provides useful insights into consumer perceptions of pre-prepared noodles in Kosovo, yet several limitations should be noted. Because data were collected through an online questionnaire, older and rural populations were underrepresented, limiting the generalizability of the results to Kosovo’s wider population. The reliance on self-reported responses may also introduce recall or judgment biases that could influence reported consumption patterns and perceptions. Some response categories (e.g., “description on the label” and “natural ingredients”) may have been interpreted similarly, and subjective terms such as “reasonable price” or “intense flavor” may vary across individuals. Although participants reported consumption based on the package they typically use, no standardized gram-based definition of portion size was provided, which may have affected portion estimates. Finally, the study reflects Kosovo’s specific cultural and economic context, and findings may not extend to other settings. Future research could address these issues by applying mixed sampling methods, standardizing portion-size measures, and conducting comparative studies across different populations.

## Figures and Tables

**Table 1:** Demographic characteristics of respondents.

Characteristics		Frequency (n = 400)	Percentage ^1^
Gender	Female	282	70.5
	Male	118	29.5
Age	Under 18	48	12.0
	18–24	130	32.5
	25–34	97	24.3
	35–44	58	14.5
	45–54	36	9.0
	Above 55	31	7.8
Education	Middle school	5	1.3
	High school	85	21.3
	Bachelor	215	53.8
	Master	83	20.8
	PhD	12	3.0
Income (euro)	<200	1	0.3
	201–400	6	1.5
	401–600	24	6.0
	601–900	93	23.3
	901–1200	173	43.2
	Above 1200	103	25.7

^1^ Percentages in the Categories (%) columns are calculated separately within each variable (gender, age, education, and income).

**Table 2:** Perceptions of pre-packaged noodles for various statements and their associations with demographic factors.

Statements	Categories (%)						Correlations ^1^					
							Gender		Education		Age	
	Strongly Disagree	Disagree	Neutral	Agree	Strongly Agree	Mean ± SD	Mann-Whitney U	*p*-Value	Kruskal-Wallis H	*p*-Value	Spearman’s Rho	*p*-Value
They are healthy.	7.4	23.2	45.2	3.4	0.8	2.59 ± 0.76	16,207.5	0.65	39.81	0.00	−0.40	0.00
Pre-packaged noodles are a less healthy alternative compared to traditional cooking.	1.8	3.2	19.8	50.0	5.2	3.67 ± 0.75	16,357.0	0.76	32.17	0.00	0.38	0.00
The salt content is low.	6.4	25.4	42.8	4.0	1.4	2.61 ± 0.78	13,462.0	0.001	42.52	0.00	−0.44	0.00
They contribute to weight gain.	1.6	4.0	37.2	32.0	5.2	3.44 ± 0.77	16,067.0	0.55	26.59	0.00	0.36	0.00
The list of ingredients on the packaging is easy to understand.	1.8	8.4	21.8	43.8	4.2	3.50 ± 0.84	14,825.5	0.06	18.378	0.001	−0.16	0.001
Frequent consumption of pre-packaged noodles may negatively affect my health.	1.2	1.8	14.4	47.4	15.2	3.92 ± 0.77	15,060.0	0.09	51.47	0.00	0.29	0.00

^1^ Responses were measured on a five-point Likert scale (1 = strongly disagree, 5 = strongly agree). Statistical tests (Mann–Whitney U, Kruskal–Wallis H, and Spearman’s rho) were applied to assess associations between perceptions and demographic factors.

**Table 3:** Reported concerns about salt, additives, and fat in pre-packaged noodles and associations with demographic factors.

Statements	Responses (%)	Correlations					
		Gender		Education		Age	
		Chi-Square	*p*-Value	Chi-Square	*p*-Value	Chi-Square	*p*-Value
Salt content	20.36	11.208	0.011	48.291	0.00	134.627	0.00
Additives and preservatives	32.05						
Fat and calories	37.14						
No, I have no concerns	10.45						

**Table 4:** Respondents’ awareness of MSG in pre-packaged noodles and associations with demographic variables.

Statements	Responses (%)	Correlations					
		Gender		Education		Age	
		Chi-Square	*p*-Value	Chi-Square	*p*-Value	Chi-Square	*p*-Value
Yes	28.5	0.408	0.523	72.775	0.00	23.508	0.00
No	71.5						

**Table 5:** Reported reasons for instant noodle consumption and their associations with age groups and gender.

Statements	Responses (%)	Correlations			
		Gender		Age	
		Chi-Square	*p*-Value	Chi-Square	*p*-Value
I don’t have time to prepare meals.	75.17	33.629	0.00	60.550	0.00
I don’t know how to cook so I choose ready-made meals.	19.54				
It’s a convenient choice on breaks.	5.29				

**Table 6:** Self-reported dependency on instant noodles and its association with consumption frequency and number of packages consumed per meal.

Statements	Responses (%)	Correlations			
		Consumption Frequency		Packages Consumed	
		Chi-Square	*p*-Value	Chi-Square	*p*-Value
Yes	33.82	321.773	0.00	194.557	0.00
No	66.18				

## Data Availability

The original contributions presented in the study are included in the article, further inquiries can be directed to the corresponding author.
